# A *Brassica napus* Reductase Gene Dissected by Associative Transcriptomics Enhances Plant Adaption to Freezing Stress

**DOI:** 10.3389/fpls.2020.00971

**Published:** 2020-06-26

**Authors:** Yong Huang, Muhammad Azhar Hussain, Dan Luo, Hongzhi Xu, Chuan Zeng, Lenka Havlickova, Ian Bancroft, Zhitao Tian, Xuekun Zhang, Yong Cheng, Xiling Zou, Guangyuan Lu, Yan Lv

**Affiliations:** ^1^ Key Laboratory of Biology and Genetic Improvement of Oil Crops, Ministry of Agriculture, Oil Crops Research Institute of the Chinese Academy of Agricultural Sciences, Wuhan, China; ^2^ Laboratory of Rapeseed, The Chongqing Three Gorges Academy of Agricultural Sciences, Chongqing, China; ^3^ Centre for Novel Agricultural Products (CNAP) M119, Department of Biology, University of York, York, United Kingdom

**Keywords:** rapeseed, associative transcriptomics, photosynthetic gas exchange parameter, tropinone reductase, alkaloid

## Abstract

Cold treatment (vernalization) is required for winter crops such as rapeseed (*Brassica napus* L.). However, excessive exposure to low temperature (LT) in winter is also a stress for the semi-winter, early-flowering rapeseed varieties widely cultivated in China. Photosynthetic efficiency is one of the key determinants, and thus a good indicator for LT tolerance in plants. So far, the genetic basis underlying photosynthetic efficiency is poorly understood in rapeseed. Here the current study used Associative Transcriptomics to identify genetic loci controlling photosynthetic gas exchange parameters in a diversity panel comprising 123 accessions. A total of 201 significant Single Nucleotide Polymorphisms (SNPs) and 147 Gene Expression Markers (GEMs) were detected, leading to the identification of 22 candidate genes. Of these, Cab026133.1, an ortholog of the *Arabidopsis* gene AT2G29300.2 encoding a tropinone reductase (*BnTR1*), was further confirmed to be closely linked to transpiration rate. Ectopic expressing *BnTR1* in *Arabidopsis* plants significantly increased the transpiration rate and enhanced LT tolerance under freezing conditions. Also, a much higher level of alkaloids content was observed in the transgenic *Arabidopsis* plants, which could help protect against LT stress. Together, the current study showed that AT is an effective approach for dissecting LT tolerance trait in rapeseed and that *BnTR1* is a good target gene for the genetic improvement of LT tolerance in plant.

## Introduction

Rapeseed (*Brassica napus* L.) is one of the major oil crops worldwide, with an average annual cropping area of 35.3 million hectares producing 72.8 million tons of seeds in the past five years (http://www.fao.org/faostat/). Meal cake, the byproduct of rapeseed is also an important source of protein-rich feed for livestock (Wanasundara et al., 2016). Due to the agronomic importance of this oil crop, there is a great interest to boost its yield *via* genetic improvement of major agronomic traits.

The winter type rapeseed is mainly grown in Europe, which requires strong vernalization and is cold tolerant (O’neill et al., 2019). However, the semi-winter type rapeseed grown in China only needs moderate or weak vernalization, and excessive exposure to low temperature (LT) stress in winter will lead to plant damage at vegetative stage and finally cause yield loss (Liao and Guan, 2001; Zhang et al., 2015; O’neill et al., 2019). Yangtze River basin is the major area for growing semi-winter rapeseed, which accounts for at least 80% of the nation’s total production (Tian et al., 2018). The rapeseed is usually sown in early October shortly after the harvest of rice in this area (Cong et al., 2019). However, in recent years, the delay of rice harvest usually lead to the postpone of rapeseed sowing until late October or early November, which results in poor germination and seedling establishment due to LT (Luo et al., 2019). The biomass of rapeseed seedling is also significantly reduced at overwintering stage, and thus is more susceptible to LT stresses, i.e. chilling (0–15°C) or freezing (<0°C) (Sage and Kubien, 2007; Zhang et al., 2012; Zhang et al., 2016). Moreover, delay of floral initiation and floral bud differentiation processes (Luo et al., 2018) and decrease of effective pod number, pod length, and seed yield (Ozer, 2003) were observed in the late-sowing rapeseed. To cope with LT stresses, plants have evolved several elaborate regulatory mechanisms; among these, balancing or coordinating the photosynthetic processes could be a critical one (Leister, 2019).

It has been established that light-harvesting complex II (LHCII) proteins in higher plants can facilitate their adaption to external biotic or abiotic environmental stresses such as drought stress or blast fungus infection (Andersson et al., 2003; Ganeteg et al., 2004; Xu et al., 2012; Liu et al., 2019b), and the positive function of LHCBs in abscisic acid (ABA) mediated signaling pathway is repressed by WARY40 (Xu et al., 2012; Liu et al., 2013). LT stress induces the accumulation of transcript encoding heat-shock proteins (HSPs), and the persistence of HSPs can enhance the chilling tolerance of tomato fruit (Sabehat et al., 1996; Ding et al., 2001). In *Arabidopsis*, HSP21 protects the photosynthetic electron transport chain against the deleterious effects imposed by heat stress (Zhong et al., 2013; Bernfur et al., 2017). However, it is unclear whether HSPs function similarly in rapeseed to alleviate injury from LT stress. The other observations have mechanistically described how the photosynthetic organisms maintain their PSII function under stressful conditions with continuing or fluctuating light (Liu et al., 2019a). For instance, the chloroplast protein HHL1 forms a complex with LQY1 to repair and re-assemble PSII, which in turn helps overcome excessive light stress (Jin et al., 2014). In rapeseed, CBF/DREB transcription factors appear to have important roles in maintaining stronger photosynthetic efficiency and higher Calvin circulating enzyme activity under LT conditions (Savitch et al., 2005).

To date, several genetic studies have been reported for quantitative trait loci (QTLs) mapping of photosynthesis (De Miguel et al., 2014; Li et al., 2014; Li et al., 2016; Liu et al., 2017; Oakley et al., 2018; Basu et al., 2019) and LT tolerance in different plant species (Jha et al., 2017). However, very few QTLs have been identified in *Brassica* species (Ge et al., 2012; Yan et al., 2015). The temperature causing 50% of the maximal damage (LT50) has been regarded as a good index for evaluating LT tolerance (Hincha et al., 1987; Steponkus et al., 1990), which is also significantly correlated with net photosynthesis rate (A_n_) in rapeseed (Urban et al., 2013). Therefore, photosynthetic gas exchange parameters such as A_n_ are a suitable index for the evaluation of LT tolerance in rapeseed that can facilitate the follow-up genetic study.

Photosynthesis plays an indispensable role in ensuring adequate energy supply throughout the plant lifecycle. Therefore, enhancing photosynthetic efficiency has been a commonly adopted strategy for crop yield improvement (Long et al., 2006; Lawson et al., 2012; Evans, 2013). In rice, a new photorespiratory bypass was assembled by over-expressing *OsGLO3*, *OsOXO3*, and *OsCATC* genes in the chloroplast, which resulted in obvious increases in photosynthetic efficiency, biomass, and grain yield (Shen et al., 2019). In rapeseed, photosynthetic efficiency has a notable effect on yield, oil content, and fatty acid composition (Ju and Li, 2012; Wang et al., 2015). However, the utilization rate of light energy in rapeseed is only 0.615% to 1.056%, which is much lower than that in rice, wheat or soybean (Zhang et al., 2017a). Therefore, it is possible to further improve the yield by enhancing photosynthetic efficiency in rapeseed.

During the long history of evolution, plants appear to overcome abrupt or mild temperature stresses in winter through a series of changes at molecular, cellular, physiological, and biochemical levels (Zhang et al., 2017b). Alkaloid is one of the major secondary metabolites that is inducible under unfavorable conditions, especially drought stress (Selmar and Kleinwachter, 2013). Heavy metals can also promote the accumulation of alkaloid in *Catharanthus roseus L.* (Srivastava and Srivastava, 2010). The short-chain dehydrogenase/reductase (SDR) protein, which belongs to the NAD(P)-binding Rossmann-fold superfamily, functions in the biosynthesis of benzylisiquinoline alkaloids (i.e. morphine, codeine) and tropane-derived alkaloids such as scopalamine, atropine, and cocaine. Tropinone reductases (TRs) are a group of SDR proteins which play key roles as a branch point in the biosynthesis pathway of tropane alkaloids (Tonfack et al., 2011). Hence, a study on TRs could help understand how alkaloids function in response to different stresses.

Associative transcriptomics (AT) strategy, which combines association mapping and transcriptome, has greatly facilitated the genetic dissection of complex traits (Bazakos et al., 2017). Considerable progress has been achieved by AT in allopolyploidy crops, such as oilseed rape and wheat, which provided a large number of causative genes or functional markers for molecular marker-assisted breeding. For instance, a transcription factor (HAG1) was identified in rapeseed by AT, which plays an indispensable role in the synthesis of aliphatic glucosinolates (Harper et al., 2012). With AT platform, the genetic studies of many other complex traits in rapeseed were also reported, including homeostasis of nitrate, phosphate, and sulfate anions (Koprivova et al., 2014), calcium and magnesium accumulation (Alcock et al., 2017), lodging resistance (Miller et al., 2018), clubroot resistance (Hejna et al., 2019), erucic acid, and tocopherol (vitamin E) isoform accumulation in seeds (Havlickova et al., 2018), and leaf nutrition concentration (Alcock et al., 2018). Recently, the power of AT was further enhanced by using a much larger panel comprising 383 rapeseed accessions (Havlickova et al., 2018). In bread wheat, two causative genes underlying stem strength variation have also been detected by AT (Miller et al., 2016). Despite the above efforts, the AT approach has not yet been applied to photosynthetic related traits under LT conditions in rapeseed.

The present study aims to identify candidate genes associated with photosynthetic gas exchange parameters including A_n_, stomatal conductance to water vapor (G_sw_), internal CO_2_ concentration (C_i_) and transpiration rate/evapotranspiration (E) in rapeseed by AT. Twenty-two candidate genes were obtained, and one was functionally validated. Ectopic expressing tropinone reductase (*BnTR1*) in *Arabidopsis* can significantly enhance transpiration rate and LT tolerance, implying its great potential for the genetic improvement of LT tolerance in plant.

## Materials and Methods

### Plant Materials

A panel comprising 123 rapeseed accessions was used for association study, which is available from the John Innes Centre, Norwich, UK (**Supplementary Table S1**) (Havlickova et al., 2018). Within this panel, there are 37 winter type, 32 spring type, 47 semi-winter type, and 7 unclassified rapeseed accessions. The panel was sown on 28^th^ Oct in 2016 in Wuhan (114.30°E, 30.57°N), China. All accessions were planted using a completely randomized block design with three replications. The temperature was recorded during the field experiments (**Supplementary Figure S1** **)**. Compared with those sown on normal occasion (28^th^ Sept in 2016), the late-sown (i.e. 28^th^ Oct) rapeseed seedlings were subjected to LT stress during winter.

### Determination of Photosynthetic Gas Exchange Parameters

The fourth true leaf of each of the 123 rapeseed accessions was chosen for the measurement of photosynthetic gas exchange parameters including A_n_, G_sw_, C_i_, and E in the open field at the 60-d-old seedling stage. Two independent plants of one accession from each block were measured by LI-6400 photosynthesis equipment (Li-Cor 6400; Li-CorInc, Lincoln, NE, USA) as described previously (Yan et al., 2015). The measurements were performed on a sunny day from 27^th^ December to 30^th^ December with a maximum temperature of 9°C in daytime and the lowest temperature of −1°C at night. The phenotypic data were collected from three blocks as biological replications (**Supplementary Table S2**). Broad-sense heritability was estimated according to a previous study (Kaler and Purcell, 2019).

The photosynthetic gas exchange parameters of *Arabidopsis* wild type (WT) and transgenic seedlings (ecotype Columbia) were measured on the second functional leaf with the light intensity of 800 μmol m^−2^ s^−1^ and CO_2_ concentration of 400 μl L^−1^. All of the *Arabidopsis* plants were grown in the greenhouse (16-h-light/8-h-dark) with the light intensity of 120 μmol m^−2^ s^−1^ at 23°C. Each leaf was measured three times as technical replications, and five independent plants from each line were measured as biological replications (**Supplementary Table S2**).

### Genome-Wide Association Study

The association panel and the procedure of AT analysis have been reported in detail previously (Havlickova et al., 2018). In brief, RNA-Seq data were generated from young leaves of the association panel harvested 21 d after sowing under 16-h-light (20°C)/8-h-dark (14°C) glasshouse conditions. The transcriptome data were mapped onto the developed ordered *Brassica* A and C pan-transcriptomes (He et al., 2015) and resulted in a set of 355,536 SNPs and RPKM values for 116,098 CDS models. Following the removal of SNP markers with minor allele frequencies below 0.01, a total of 256,397 SNPs were retained (http://www.yorknowledgebase.info/) and used as marker input for the Associative Transcriptomics analysis as previously described (Harper et al., 2012; Lu et al., 2014). The current study adopted a compressed mixed linear model including both fixed and random effects according to a previous method (Lipka et al., 2012). The *P*-values (–log_10_ converted) for all SNPs were plotted against their physical position in the “pseudo-molecules” to produce a Manhattan plot. The Bonferroni significance threshold was set as *P* = 3.9 × 10^−6^ (1/256397) (Duggal et al., 2008). Allelic effects of all candidate SNPs were calculated according to a previous study (Prado et al., 2017); a positive effect indicates that the allele increases the trait value, whereas a negative effect indicates that the alternative allele increases the trait value.

The transcript level was quantified as reads per kb per million aligned reads (RPKM) across the panel. After filtering (RPKM ≤ 0.4), a total of 53,889 gene expression marker (GEM) was obtained. The GEM was regarded as the dependent variable and trait data as the independent variable (Wood et al., 2017). The fixed effect linear model was performed to assess the relationship between gene expression level and the traits (Alcock et al., 2017). The *P*-value for each GEM was converted (-log_10_ *P*) and plotted against its physical position to generate a Manhattan plot. The Bonferroni significance threshold was set at *P* = 1.85 × 10^−5^ (1/53889).

### Growth Conditions and Stress Treatments

To determine the gene function of *BnTR1* on *Arabidopsis* under freezing conditions, the seeds of WT and transgenic *Arabidopsis* plants were germinated on 1/2 MS medium and 1/2 MS medium plus 30 μg/ml hygromycin, respectively. After 1 week growth at 23°C (16-h-light/8-h-dark), healthy seedlings with uniform sizes were transplanted into 8×8 cm pots (four plants per pot). Then, the 21-d-old plants were transferred into a growth chamber at −4°C for 4 h after 24 h of cold acclimation and recover at 23°C (16-h-light/8-h-dark). Six pots from each transgenic line or WT plants were used to investigate the survival rate 3 d after recovery. The leaves were sampled for physiological and biochemical measurements.

To determine the effects of alkaloid on *Arabidopsis* and rapeseed seedlings, WT plants of *Arabidopsis* were firstly grown under the same conditions as above. Before the freezing treatment, a dosage of 0, 10, and 30 nmol alkaloid (atropine) per seedling was added to the soil for *Arabidopsis*, while a dosage of 0, 50, and 150 nmol per seedling was added to the soil for rapeseed accession Zhongshuang 11 (ZS11) according to the previous study (Hara and Kurita, 2014). After inoculation overnight, the 21-d-old *Arabidopsis* plants were transferred into a growth chamber at −4°C for 4 h, the three-leaf-stage rapeseed plants were transferred into the growth chamber at −4°C for 4 h (Yan et al., 2019). The survival rate was investigated in six pots 3 d after recovery (**Supplementary Table S2**).

To investigate the expression of candidate genes under LT stress conditions, six rapeseed accessions showing extreme photosynthetic efficiency (i.e. Sv706118, Kajsa, Callypso, Libritta, Gefion, and Jupiter; **Supplementary Table S1**) were used for expression analysis of candidate genes. The four-leaf seedlings were transferred into the plant growth chamber with −4°C for 4 h (Yan et al., 2019). The leaves were sampled before, and after freezing treatment, and then used for RNA extraction and molecular analysis. The detailed information was listed in **Supplementary Table S2**.

### Physiological and Biochemical Measurements

To determine the effects of *BnTR1* on *Arabidopsis* at physiological and biochemical levels under LT stress conditions, the seedlings of transgenic and WT *Arabidopsis* plants were treated under freezing conditions (−4°C for 4 h) and, the leaves of 21-d-old seedlings were sampled at three time-point (i.e. before freezing treatment, after freezing treatment, and recovery for 3 d at 23°C) for measuring physiological and biochemical characteristics, including Fv/Fm, electrolyte leakage, 3,3′-diaminobenzidine (DAB) staining, proline content, soluble sugar content, reactive oxygen species (ROS) scavenging enzymes activity, H_2_O_2_ content, alkaloid content. All measurements were performed with at least three biological replications; detail information for experimental design and plant materials used was listed in **Supplementary Table S2**.

The Fv/Fm measurement was performed using the second functional leaves before and after the freezing treatment. The leaves were firstly immersed in 1% agarose overnight avoiding of the dark, then the chlorophyll measurement (Fv/Fm) was measured using the modulated chlorophyll fluorescence instrument (PAM-2500; Walz) as previously reported (Lv et al., 2017).

The electrolyte leakage measurement was performed according to the previous study (Lv et al., 2016). Briefly, six leaves from six were cut and immersed in 8 ml of double-distilled H_2_O in a 10-ml tube. After shaking overnight, the electrolyte leakage was measured using a model DDS-IIA device (Leici Instrument) as R1; it was measured again and recorded as R2 after boiling at 95°C in a water bath for 15 min and cooling down. The relative electrolyte leakage was calculated as a ratio of R1/R2.

The proline and soluble sugar contents were measured using the kits from Beijing Solarbio Science & Technology as described before (Yan et al., 2019). In brief, 0.1 g fresh tissue was powdered and incubated in 1 ml 3% sulfosalicylic acid (for proline) or ddH_2_O (for soluble sugar). After centrifuging, 400 μl supernatant was mix with other reaction buffers and incubated at 95°C in a water bath for 15 min, then the absorbance was measured using MULTISCAN FC (Thermo Scientific).

The ROS scavenging enzymes activity was measured by commercial kits according to the manufacturer’s instruction (Beijing Solarbio Science & Technology) with minor modification (Yan et al., 2019). 0.1 g fresh tissue was powdered using 1 ml 0.05 mol/L PBS buffer (pH 7.8). The supernatant was obtained after centrifuging at 8,000*g* for 10 min at 4°C and used for superoxide dismutase (SOD) activity, peroxidase (POD) activity, catalase (CAT) activity measurement using MULTISCAN FC (Thermo Scientific).

DAB staining was performed as previously described (Ning et al., 2010; Zhang et al., 2011). The fourth functional leaf of each plant was sampled and infiltrated in 0.1 mg/ml 3,3′-diaminobenzidine liquid (50 mM Tris-acetate buffer, pH 5.0). After incubation overnight at 25°C in the dark, the stained leaves were photographed after removing the chlorophyll by absolute ethanol. The H_2_O_2_ content was quantified according to the instruction of the kit (Beijing Solarbio Science & Technology) (Yan et al., 2019).

The total alkaloid was extracted as described previously (Zhang et al., 2004; Chen et al., 2013). The freeze-dried leaves were powdered; 0.1 g powder was homogenized overnight with 1.0 ml 70% aqueous methanol at 4°C. Following centrifugation at 10,000 g for 10 min at 4°C, the extracts were absorbed (CNWBOND Carbon-GCB SPE Cartridge, 250 mg, 3 ml; ANPEL) and filtrated (SCAA-104, 0.22 μm pore size; ANPEL). Next, the total alkaloid content was determined using the alkaloid ELISA kit (Hiton) according to the instructions.

### RNA Extraction and Gene Expression Analyses

Total RNA was extracted from the *Arabidopsis* or rapeseed seedlings with TransZol reagent (Trans) and converted to the first-strand cDNA using the EasyScript^®^One-Step cDNA Synthesis SuperMix (Trans). The Quantitative Real-time PCR (qRT-PCR) was performed using Power SYBR^®^Green PCR Master Mix according to the manufacturer’s instructions on a StepOnePlusReal-Time PCR System (Applied Biosystems). The primers used for expression analysis of candidate genes either detected by AT approach or involved in the known biosynthetic pathway of alkaloid were listed in **Supplementary Table S3**. The relative expression level was determined as previously described (Livak and Schmittgen, 2001).

### Vector Construction and Gene Transformation

To generate transgenic lines over-expressing candidate gene (*BnTR1*), the coding sequence (CDS) of *LOC106445422* was amplified from a rapeseed variety ZS11 and ligated into vector pCAMBIA1300 driven by a tobacco cauliflower mosaic virus 35S promoter (CaMV35S). The construct was introduced into *Arabidopsis* variety Columbia (Col) by *Agrobacterium*-mediated transformation (Zhang et al., 2006). Three T4 homozygous lines (L1, L3, L5) significantly over-expressing *BnTR1* were obtained by screening at 30 μg/ml hygromycin. The primers used for vector construction were listed in **Supplementary Table S3**.

### Statistical Analyses

Statistical analyses were conducted with Microsoft Excel (2003) and SPSS (version 22.0) software using one-way analysis of variance (ANOVA) with Tukey’s multiple comparisons test or two-tailed Student’s *t*-test. All data were presented as the means ± standard error (SE) based on three replicates. *P*<0.05 and *P*<0.01 were considered statistically significant and highly significant, respectively.

## Results

### Phenotypic Variation of Photosynthetic Gas Exchange Parameters

**Figure 1:**
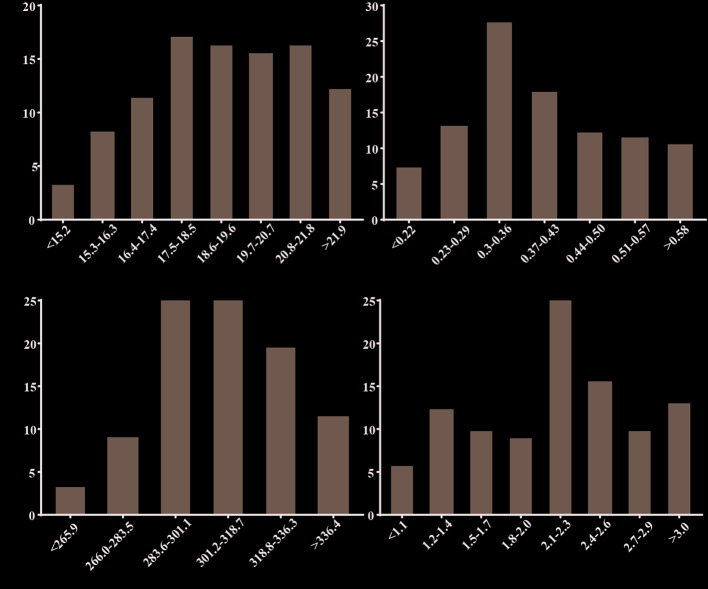
Phenotypic variation of photosynthetic gas exchange parameters in 123 rapeseed accessions. Trait definition: Net photosynthesis rate (A_n_), Stomatal conductance to water vapor (G_sw_), Internal CO_2_ concentration (C_i_), Transpiration rate (E).

A field-grown rapeseed association panel experienced long-term LT stress in winter (**Supplementary Figure S1**). The photosynthetic gas exchange parameters such as A_n_, G_sw_, C_i_, and E were measured since they can reflect photosynthetic efficiency for a plant. Substantial variations for the four traits were observed in 123 rapeseed accessions (**Figure 1**). A_n_ varied from 12.87 to 24.02 with a mean of 19.29 μmol (CO_2_) m^−2^ s^−1^. Similarly, G_sw_ ranged from 245.21 to 383.28 μmol (CO_2_) mol^−1^ and E from 0.96 to 3.75 mmol (H_2_O) m^−2^ s^−1^. G_sw_ was the most variable trait since it has the largest coefficient of variation (0.69), with a minimum of 0.16 and a maximum of 2.53 mol (H_2_O) m^−2^ s^−1^; the range of broad-sense heritability varied from 49.49% to 68.91% (**Supplementary Table S4**). Moreover, the four traits in the association panel were positively correlated with each other (*P* ≤ 0.01) (**Supplementary Table S5**). For instance, there was a strong correlation between A_n_ and E (*r* = 0.785, *P*<0.01).

### Associative Transcriptomics for Photosynthetic Gas Exchange Parameters

**Figure 2:**
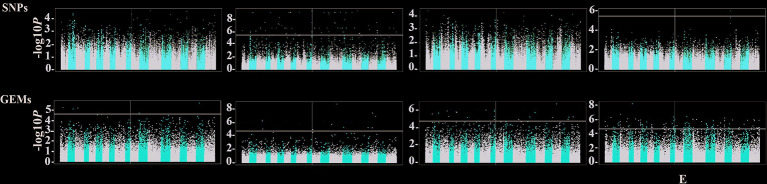
Manhattan plots for AT analysis in 123 rapeseed accessions. Manhattan plots from left to right, represented for A_n_, G_sw_, C_i_, and E using SNPs (upper section) and GEMs (bottom section), respectively. The -log10 (*P* values) were plotted against the position of the SNPs or GEMs on 19 chromosomes of *Brassica napus*. The black line represents the -log10 (*P* values) converted Bonferroni significance threshold for SNP (5.41) and GEM (4.73), respectively.

To identify genomic regions controlling photosynthetic related traits, AT was performed in rapeseed. Using the mixed linear model, a total of 201 significant SNPs were detected, which originated from 148 CDSs (**Figure 2**). Unexpectedly, most of the significant CDSs were associated with G_sw_ trait, while only one was related to E trait. The detail results including physical positions, *P*-values, allelic effects were summarized in **Supplementary Table S6**. For GEM analysis, a total of 145 CDSs above the corrected Bonferroni thresholds were identified (**Figure 2**). Of these, a respective of 5, 10, 20, and 110 CDSs were detected for A_n_, G_sw_, C_i_, and E. The detailed information, including physical positions and *P* values for all significant GEMs, was listed in **Supplementary Table S7**.

**Figure 3:**
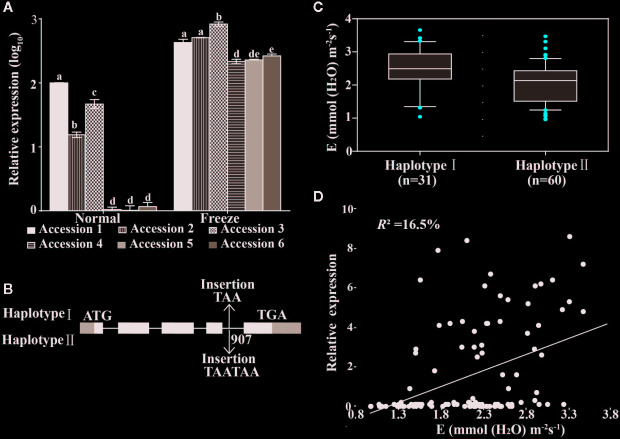
Expression and allelic variation of *BnTR1* in rapeseed. **(A)** Expression analysis of *BnTR1* (homolog of *Cab026133.1*) in six accessions corresponding to Hap 1 and Hap 2 under freezing conditions. The name of accessions 1 to 6 was Sv706118, Kajsa, Callypso, Libritta, Gefion, and Jupiter, respectively. *ACTIN* gene was used as an internal control. Bars indicate the SE of three biological replicates. Different letters indicate significant differences at *P*< 0.05 (one-way ANOVA with Tukey’s multiple comparisons test). **(B)** Correlation analysis between Transpiration rate (E) value and expression level of *Cab026133.1* in the association panel (n=123). *R^2^* indicates the coefficient of determination in linear regression. **(C, D)** Allelic variations at *BnTR1* formed two main haplotypes and their effects on E value.

By annotating all the above significant CDS in public database TAIR (https://www.arabidopsis.org/), the present study shortlists the number of candidate genes to only 22 that putatively involved in photosynthesis or LT stress response (**Supplementary Table S8**). To verify their roles in rapeseed with or without LT stress, six accessions from the association panel were selected; Sv706118, Kajsa, and Callypso (Accessions 1–3) exhibited higher photosynthesis efficiency and were tolerant to freezing stress, while Libritta, Gefion, and Jupiter (Accessions 4–6) presented lower photosynthesis efficiency and were sensitive to freezing stress (**Supplementary Table S1**, **Supplementary Figure S2**). The gene expression profiles were investigated by qRT-PCR in the six accessions under freezing conditions. Results showed that all of these genes exhibited a significantly different expression level in the six accessions, suggesting that these genes indeed involved in freezing stress response. *Cab026133.1* seemed to have a much higher expression level in the three tolerant accessions (Accessions 1–3) with or without freezing stress (**Figure 3A**); so did *Cab011968.1*, *Cab022014.2*, and *Cab007526.2*, an ortholog of inorganic carbon transport protein (AT1G70760.1), bZIP transcription factor (AT5G28770.3) and citrate synthase 2 (BnaA10g24440D), respectively. An opposite trend was observed for *Bo5g017460.1* and *Cab008128.1*, an ortholog of F-box family protein (AT2G32560.1) and dehydroascorbate reductase (AT5G16710.1), respectively (**Supplementary Figure S3** *).*

### Selection and Characterization of Candidate Gene

Cab026133.1 was selected for further analysis because it not only exhibited the highest *P*-value (6.33 × 10^−9^) for E trait in GEM analysis (**Supplementary Table S8**) but also highly expressed in LT tolerant accessions (**Figure 3A**). Besides, the expression of *Cab026133.1* (presented as RPKM) across the rapeseed panel was positively correlated with E level (*r*=0.406, *P* < 10^−3^) and accounted for 16.5% of trait variation (**Figure 3B**). The ortholog of Cab026133.1 in *Arabidopsis* (AT2g29300) encodes an SDR protein involved in the oxidation-reduction process of secondary metabolites, such as phenols, isoprene, and alkaloid (Selmar and Kleinwachter, 2013). SDR proteins are classified into six subfamilies, and the tropinone reductase subfamily belongs to the major route of alkaloid biosynthesis (Tonfack et al., 2011). The alignment of amino acid sequence clearly illustrated that LOC106445422 shared 87.3% similarity with Cab026133.1 and 57% with CoTR, a known tropinone reductase in *Cochlearia officinalis* (Brock et al., 2008). LOC106445422 displayed typical SDRs motifs (Gly-X_3_-Gly-X-Gly) and four conserved residues that form the catalytic tetrad NSYK (N127, S155, Y168, K172) (**Supplementary Figure S4**). The current study name LOC106445422 as *BnTR1* and used as the candidate gene for the follow-up studies.

To assess the effect of allelic variation on *BnTR1* in the rapeseed association panel, the genomic region covering the whole gene as well as the 2-kb promoter region was amplified. One TAA/TAATAA insertion was detected in the fourth intron that formed two major haplotypes, i.e. Haplotype I (with TAA insertion) and Haplotype II (with TAATAA insertion) (**Figure 3C**). Haplotype I (n=31) displayed significantly higher E value than Haplotype II (n=60) (*P*=5.43 × 10^−4^) (**Figure 3D**). Besides, *the LT tolerant* Accessions 1 to 3 were determined as Haplotype I while sensitive Accessions 4 to 6 as Haplotype II at BnTR1 locus (**Supplementary Table S1**). The expression level of *BnTR1* in Accessions 1 to 3 was also significantly higher than that in accessions 4 to 6 with or without stress treatment (**Figure 3A**). Therefore, it was evident that E variation may be attributed to expression or allelic variation at *BnTR1*, which was expressed almost in all tissues of rapeseed at both vegetative and reproductive stages (**Supplementary Figure S5**).

### Ecotopic Expressing *BnTR1* Enhances Freezing Tolerance in *Arabidopsis*

**Figure 4:**
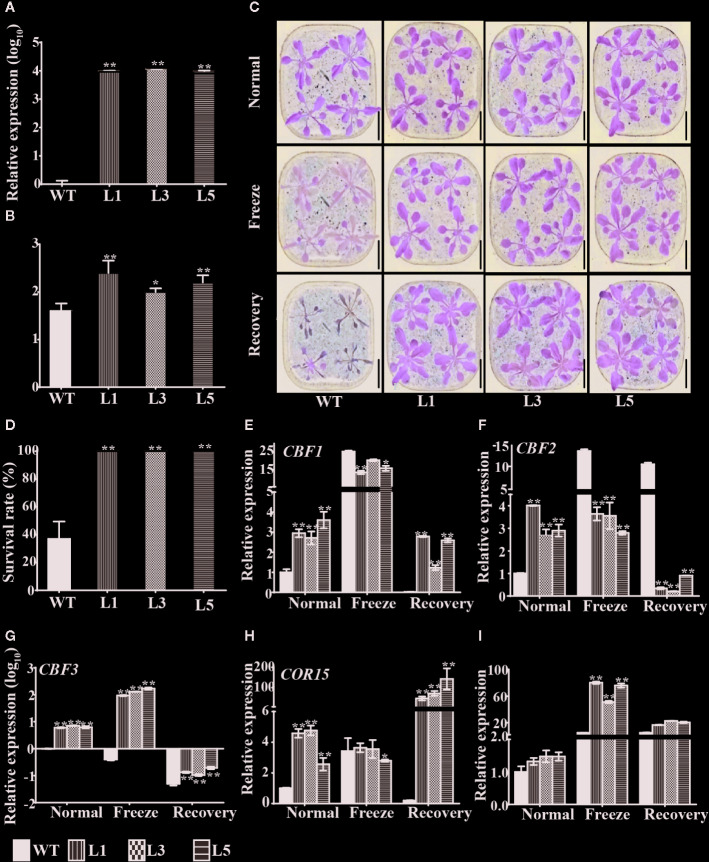
BnTR1 confers freezing tolerance in *Arabidopsis*. **(A)** Expression analysis of the *BnTR1* transgenic plants (L1, L3, L5) and WT plants under normal condition (i.e. 23°C). **(B)** Investigation of transpiration rate **(E)** value in the *BnTR1* transgenic lines and WT plants under normal conditions. **(C)** Performance of the transgenic lines and WT plants before and after freezing treatment (−4°C for 4 h). Scale=2 cm. **(D)** Survival rates of the transgenic lines and WT plants after freezing treatment. **(E**–**I)** Relative expression levels of *CBF1* **(E)**, *CBF2* **(F)**, *CBF3* **(G)**, *COR15* **(H)**, *RD29A* **(I)** in the transgenic lines and WT plants before and after freezing stress with the *Arabidopsis ACTIN* gene used as an internal control. Normal represents 23°C, freezing treatment represents 4 h at −4°C, recovery represents 3 d of recovery at 23°C. Bars indicate the SE of three biological replicates. Significant differences are determined by Student’s *t-*test (*P < 0.05, or **P < 0.01).

To analyze gene function, three independent *Arabidopsis* lines (L1, L3, L5) ectopic expressing *BnTR1* were generated. All transgenic lines showed an increased expression level of *BnTR1* in comparison to the WT plants (**Figure 4A**). At the seedling stage, E value of the transgenic lines was much higher than that of WT plants (**Figure 4B**), thus confirming that BnTR1 controls transpiration rate. Since photosynthetic gas exchange parameters are significantly correlated with cold tolerance (Urban et al., 2013), it was speculated that BnTR1 also involves in LT stress. To test this hypothesis, seedlings were treated at −4°C for 4 h and then recover at 23°C. All of the transgenic lines were shown to be freezing-tolerant because there was no obvious syndrome, while the WT plants were wrinkled and hydrophanous (**Figure 4C**). After recovery for 3 d at 23°C, all transgenic plants survived, but 62% of the WT plants died (**Figure 4D**). These results strongly suggested that BnTR1 enhanced the freezing tolerance of *Arabidopsis* plants.

To assess whether C-repeat-binding factors (CBFs) contributed to the enhanced freezing tolerance of the transgenic lines, the current study measured the relative expression level of *CBFs* in transgenic and WT plants after freezing stress (**Figures 4E**–**G**). In the transgenic lines grown under normal conditions, expressions of *CBFs* were much higher than that in WT plants, indicating that they were markedly induced by *BnTR1*. Moreover, the expression of *CBF1* and *CBF3* was significantly up-regulated and *CBF2* down-regulated by *BnTR1* during freezing treatment in comparison with those of normal conditions. The current study further examined the expression level of CBFs-targeted cold-responsive genes (COR genes) (**Figures 4H, I** **)**. As expected, the transcript levels of COR genes (namely *COR15* and *RD29A*) in the transgenic lines were highly induced when compared with that in WT plants. These results indicated that *BnTR1* influences CBF regulon in the stress-signaling pathway to control freezing tolerance.

**Figure 5:**
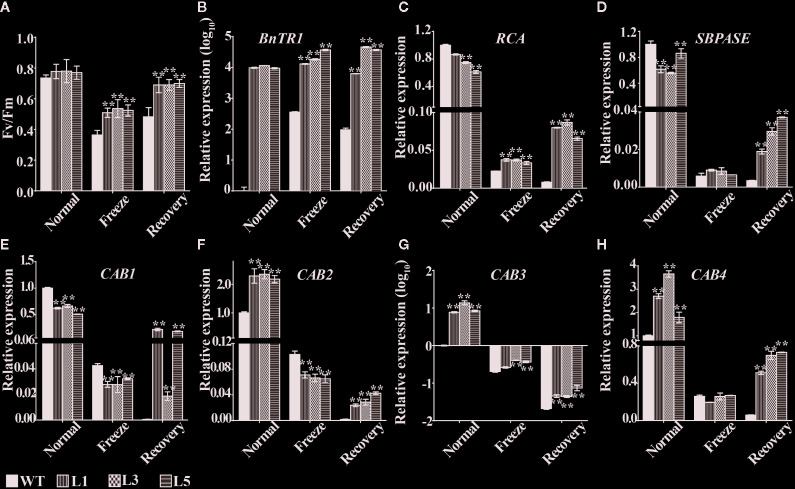
Variation of photosynthetic related traits and genes expression pattern in *BnTR1* transgenic plants. **(A)** Fv/Fm ratio in the transgenic lines and WT plants under freezing stress conditions. **(B–H)** Relative expression levels of *BnTR1* **(B)**, *RCA* **(C)**, *SBPASE* **(D)**, *CAB1* **(E)**, *CAB2* **(F)**, *CAB3* **(G)**, *CAB4* **(H)** in the transgenic lines and WT plants before and after freezing stress treatment with *Arabidopsis ACTIN* gene used as an internal control. L1, L3, L5 represent three independent homozygous lines of *BnTR1* transgenic plants. Bars indicate the SE of three biological replicates. Significant differences are determined by Student’s *t-*test (*P < 0.05, or **P < 0.01).

The chlorophyll fluorescence parameter Fv/Fm, an indicator for the potential maximum photosystem II (PSII) capacity of plants, has been widely used to determine the ability of tolerance to environmental stresses under laboratory conditions (Mishra et al., 2014; Thalhammer et al., 2014). Here, markedly higher Fv/Fm ratio was observed in leaves of the transgenic lines under freezing as well as normal conditions (**Figure 5A**). To further clarify whether the difference of photosynthetic capacity was caused by the excessive expression of *BnTR1*, the current study assessed the expression level of *BnTR1* and other genes involved in photosynthetic processes such as *RCA*, *SBPASE* (for CO_2_ fixation or assimilation) and *CAB1-4* (for light-harvesting) (Sun et al., 2017; Basu et al., 2019) (**Figures 5B**–**H**). Under normal conditions, the expression level of *RCA*, *SPASE,* and *CAB1* were slightly reduced in the transgenic lines compared to WT plants, whereas CAB2, CAB3, and CAB4 were induced. The freezing treatment led to a notable suppression of all genes; however, the expression of genes in the transgenic lines returned to a high level when freezing stress was removed.

### BnTR1 Contributes to Cell Membrane Protection and Antioxidants

**Figure 6:**
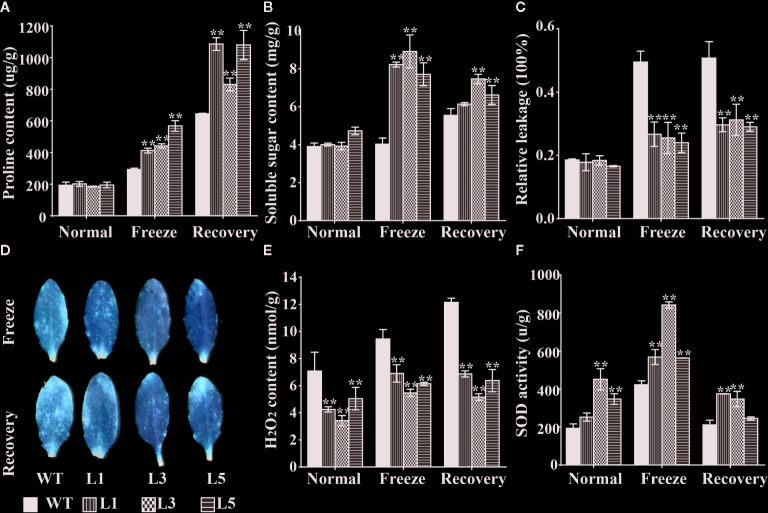
Physiological characterization of *BnTR1* transgenic plants under freezing stress conditions. **(A**–**F)** Investigation of the proline content **(A)**, soluble sugar content **(B)**, relative leakage **(C)**, DAB staining analysis **(D)**, H_2_O_2_ content **(E)**, SOD activity **(F)** in the transgenic lines and WT plants under freezing stress conditions. L1, L3, L5 represent three independent homozygous lines of *BnTR1* transgenic *Arabidopsis* plants. Bars indicate the SE of three biological replicates. Significant differences are determined by the Student’s *t-*test (*P< 0.05, or **P < 0.01).

Altering the osmotic balance to maintain the integrity and stability of cell membrane is proposed to be an efficient way for plants adapting to the changing environments (Morsy et al., 2005; Valerio et al., 2011). To test this hypothesis, physiological and biochemical assays were carried out. Results showed that freezing treatment led to only 40% to 200% increase of proline content in WT plants but as high as 100% to 300% in transgenic plants, indicating that the transgenic plants expressing *BnTR1* could accumulate more proline (**Figure 6A**). The soluble sugar content showed a similar pattern (**Figure 6B**). However, the electrolyte leakage increased more rapidly in WT plants than in transgenic lines (**Figure 6C**). These results implied that BnTR1 actively responded to freezing stress by maintaining cell membrane stability and osmotic balance.

Antioxidants, which function in scavenging the reactive oxygen species (ROS), are generally considered as another effective element in defending abiotic stresses (Choudhury et al., 2017). To determine whether *BnTR1* affects the antioxidant system, the accumulation of ROS was determined by DAB staining. The brown precipitate (H_2_O_2_) in WT was much larger than that in the transgenic lines (**Figure 6D**), indicating that WT plants had a higher level of H_2_O_2_ content than transgenic plants (**Figure 6E**). Oxidoreductases like POD, SOD, and CAT also function in scavenging redundant ROS (Gupta et al., 2016). Here, we found that the transgenic lines exhibited stronger SOD activity than WT (**Figure 6F**), which help plants alleviate oxidation damage from freezing conditions. However, no significant difference was detected for POD and CAT activities. Together, the enhanced freezing adaption for transgenic plants could be attributed to the increased ROS scavenging ability.

### BnTR1 Positively Affects Alkaloid Metabolism

**Figure 7:**
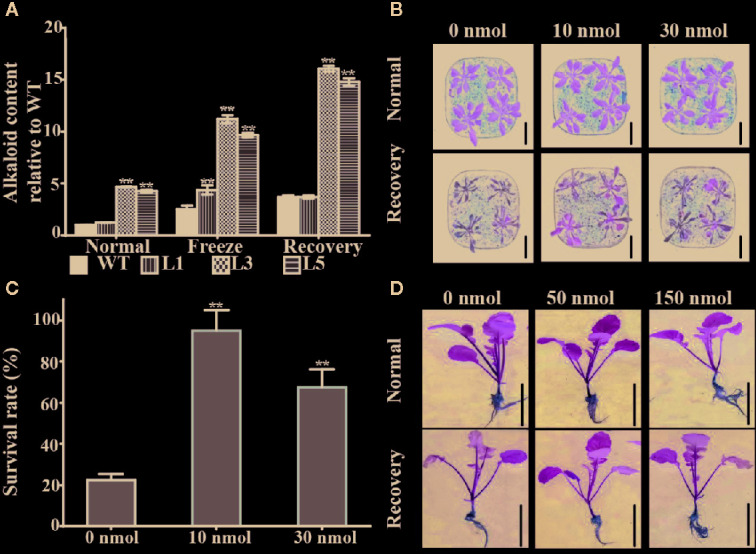
BnTR1 mediates alkaloid accumulation and exogenous atropine application enhances freezing tolerance. **(A)** Total alkaloids accumulation in *BnTR1* transgenic lines and WT plants under freezing stress conditions. L1, L3, L5 represent three independent homozygous lines of *BnTR1* transgenic *Arabidopsis* plants. **(B)** Phenotypes of *Arabidopsis* WT plants with exogenous atropine application (0 nmol per plant, 10 nmol per plant, 30 nmol per plant) under freezing stress conditions. Scale = 2 cm. **(C)** Survival rates of *Arabidopsis* WT plants with exogenous atropine application after the freezing treatment. **(D)** Phenotypes of rapeseed WT plants with exogenous atropine application (0 nmol per plant, 50 nmol per plant, 150 nmol per plant) under LT conditions. Scale=5 cm. Bars indicate the SE of three biological replicates. Significant differences are determined by Student’s *t-*test (*P < 0.05 or **P < 0.01).

BnTR1 (homolog of AT2g29300) is predicted to be a tropinone reductase involved in the biosynthesis of alkaloid (KO00960), which mainly produces atropine. Although all alkaloids (with more than 12,000 different structures) have been well-documented in pharmacology, their roles in abiotic stress remain elusive (Schlager and Drager, 2016). To investigate the specific role of BnTR1 in alkaloid metabolism, the total alkaloids content was quantified. As expected, the transgenic lines led to one- to two-fold increase of alkaloids contents compared with WT plants after freezing stress (**Figure 7A**). To further confirm the effect of alkaloid on stressed plants, exogenous atropine was applied to WT plants, since atropine was considered to be the product of alkaloid metabolism (KO00960) (Hara and Kurita, 2014). Results demonstrated that application of 10 nmol atropine per plant significantly rescued the susceptibility of WT plants, but the protective effect was weakened when dosage increase to 30 nmol (**Figure 7B**). The survival rate increased by three- to four-fold compared with WT plants without atropine treatment (**Figure 7C**).

To further elucidate the protective role of alkaloid in rapeseed, the current study applied exogenous atropine to a widely cultivated rapeseed variety, ZS11, under freezing conditions. Phenotypic analysis showed that the wilting phenotype of ZS11 plants was partially rescued by exogenous atropine application (50 and 150 nmol per plant) (**Figure 7D**), while no significant difference was observed in the survival rate. It was concluded that alkaloids alleviated the damage on plants from extreme LT stress.

## Discussion

### Power of AT Approach

*Brassica napus* originated from the hybridization of *Brassica rapa* and *Brassica oleracea* which contribute the A and C genomes, respectively (Cheung et al., 2009). There is only 15% difference in nucleotide structure and 3% difference in transcriptional expression patterns between chromosomes A and C, which limits the development of SNP markers in genome-wide association analysis until the availability of the high throughput next-generation sequencing technology (Adams et al., 2003; Higgins et al., 2012). Over the past years, AT approach based on abundant SNP markers and GEMs, has successfully simplified the complexity of the whole genome (Harper et al., 2012), and has been widely applied in rapeseed, wheat, and other polyploidy crops (Harper et al., 2012; Schuster et al., 2013; Koprivova et al., 2014; Lu et al., 2014; Harper et al., 2016a; Harper et al., 2016b; Miller et al., 2016; Alcock et al., 2017; Havlickova et al., 2018; Miller et al., 2018). However, the genetic basis of photosynthetic-related traits in oil crops remains elusive. Here, the genetic architecture of photosynthetic gas exchange parameters was investigated by AT approach, and a gene termed *BnTR1* was confirmed to be responsible for E trait (**Figure 4**), which might be a promising candidate beneficial to rapeseed in coping with climatic changes. Several other interesting candidates were also identified. For instance, Bo5g155110.1 was found to be significantly associated with E traits (*P*=1.1×10^−5^), and down-regulated by freezing stress (**Supplementary Figure S3**). The homolog in *Arabidopsis* is Cyclophilin38 (AtCYP38), which functions in the assembly and maintenance of PSII super complex (**Supplementary Table S7**). The loss-of-function mutant of *AtCYP38* shows reduced growth rate and photosynthetic efficiency compared to its wild type. Additionally, the D1 and D2 proteins in PSII reaction center show a short half-life, resulting in susceptibility upon exposure to excessive light (Fu et al., 2007; Sirpio et al., 2008). *Bo3g153100.1* was hit by an SNP marker, with -log10 (P-*value)* value as high as 9.06 (**Supplementary Table S6**), it was also markedly up-regulated by freezing stress (**Supplementary Figure S3**). Bo3g153100.1 was homologs to AT4G37930 in *Arabidopsis*, which has been documented in the photorespiration process (Takahashi et al., 2007). In the knockout mutant of AT4G37930, the photorespiration pathway is destroyed, and the chlorophyll deficiency results in chlorosis (Voll et al., 2006). Therefore, it seems that AT is a powerful tool to identify candidate genes for photosynthesis and LT stress in rapeseed. It is worthy to further study the function of all 22 candidate genes identified here.

### The Positive Role of BnTR1 Under LT Conditions

It is generally accepted that photosynthesis is vulnerable to adverse environmental stresses such as extreme temperature, salinity, drought or combined stresses (Sainz et al., 2010; Strzepek et al., 2019). Abiotic stresses lead to photoinhibition as well as excessive generation of ROS, which suppresses the photosynthetic progress and ultimately repress the growth and productivity in plants (Gabriel et al., 2010; Nishiyama et al., 2014). During the long-term evolution, plants have developed a variety of adaptive mechanisms to cope with the stressful conditions (Liu et al., 2019a; Strzepek et al., 2019). The CBF transcription factors in rapeseed are known to be responsible for the photosynthetic performance; CBF5 and CBF17 enhance the energy conversion efficiency under LT conditions (Savitch et al., 2005; Dahal et al., 2012). CBF1-CBF3, also termed dehydration-responsive element-binding factors, have been well-documented in plants. In *Arabidopsis*, CBF2 represents a negative regulator for LT response, while CBF1 and CBF3 are positive regulators (Novillo et al., 2004; Novillo et al., 2012). Interestingly, increased expression of *CBF1* and *CBF3* and repressed expression of *CBF2* were observed in the *BnTR1* transgenic lines (**Figures 4E–G**), indicating that BnTR1 represented a unique influence on CBF members. Both alleviated accumulation of ROS and activated SOD enzyme system was observed in the *BnTR1* transgenic lines (**Figure 6**), suggesting active impacts of BnTR1 on the ROS scavenging system. In addition, ectopic expressing *BnTR1* also promote the expression of the genes associated with plant photosynthesis (**Figure 5**). Specifically, the decrease of *RCA* transcripts leads to lower A_n_ value, which in turn slows down plant growth (Von Caemmerer et al., 2005; Yin et al., 2010). Moreover, RCA enhances growth and photosynthesis under moderate heat stress conditions (Kurek et al., 2007; Kumar et al., 2009). However, overexpression of *SBPASE* improves sugar accumulation and enhanced photosynthesis efficiency (Miyagawa et al., 2001; Feng et al., 2007; De Porcellinis et al., 2018). In the present study, PSII was severely repressed during freezing treatment, whereas the *BnTR1* transgenic *Arabidopsis* plants still exhibited higher Fv/Fm level compared to WT (**Figure 5A**). These observations suggest that BnTR1 triggered a series of responses including the ROS scavenging system, CBF pathway, and photosynthetic processes. However, further work is required to confirm their roles in LT tolerance.

### Protective Role of Alkaloids Under LT Conditions

Previous studies have been instrumental in revealing some metabolites underlying stress response mechanisms (Thalmann and Santelia, 2017). So far, definitions of alkaloids are generally focused on strong pharmacological effects, such as antimitotic, antidote, anticancer, and antioxidants (Schlager and Drager, 2016). The concentration of alkaloid compounds is predominantly inducible when plants are subjected to multiple stresses (Srivastava and Srivastava, 2010; Cheng et al., 2018). However, few studies have recognized the positive correlations between alkaloids and stress resistance. Application of sanguinarine for *Arabidopsis* seedlings under heat stress condition could markedly enhance the tolerance, which presumably by promoting the expression of heat shock proteins like HSP70 and HSP90.1S (Hara and Kurita, 2014; Matsuoka et al., 2016). BnTR1 is predicted to encode a tropinone reductase, which is involved in the metabolic pathway of atropine alkaloids. The current study determined the total alkaloids content under stress conditions, which showed an increased level in *BnTR1* transgenic lines under normal and freezing stress conditions compared with WT plants (**Figure 7A**). Moreover, the application of exogenous atropine alleviated the damage caused by extreme temperature in both *Arabidopsis* and rapeseed seedlings (**Figures 7B, D** **)**, which was in agreement with the observations in sanguinarine under heat stress conditions (Schlager and Drager, 2016). However, more studies are still required to confirm that alkaloids could function as a protectant for plants to confer stronger resistance to LT stresses. The current study has compared the expression level of stress-related genes in *Arabidopsis* plants treated with exogenous atropine under freezing conditions (**Supplementary Figure S6**). It was found that atropine could promote the expression of *CBF1*, *CBF3*, *CAB1*, *CAB3*, *CAB4*, *SPASE* before or after freezing treatment, but the extent is much lower than that induced by BnTR1 (**Figures 4** and **5**). Thus the results confirmed at least in part the protective role of atropine for a plant in adaption to LT stress. It is proposed that BnTR1 works as an effecter *via* metabolizing alkaloids accumulation, photosynthesis, CBF/DREB pathways, and ROS scavenging system in stressed *Arabidopsis*, which in turn contributes to the adaptation under LT conditions.

## Conclusions

During overwintering for the semi-winter type rapeseed grown in China, the extremely low temperature has a deleterious impact on plant productivity. Therefore, the identification of genes responsible for stress response is the prime interest of researchers. Despite of limited phenotypic data, our associative transcriptomics approach has been successfully used to dissect the genetics of photosynthetic-related traits under low temperature conditions. The first short-chain dehydrogenase/reductase, BnTR1 was identified in rapeseed, which improved the transpiration rate and freezing tolerance of *Arabidopsis* plants. Taken together, our findings illustrated the molecular mechanism of plant adaption to low temperature stress. Finally, this work sheds light on the way to increase low temperature tolerance in rapeseed by genetic engineering strategies.

## Data Availability Statement

The original contributions presented in the study are included in the article/supplementary material; further inquiries can be directed to the corresponding authors.

## Author Contributions

Figures, study design: YH, MH. Investigation and data collection: YH, HX, CZ. Data analysis: GL, ZT, LH, IB. Validation: DL. Resources: LH, IB. Writing—original draft: YH, MH. Writing—review and editing: YL, GL. Funding acquisition: XZh, YC, XZ, GL, YL.

## Funding

This research was financially supported by funds from the National Key Research and Development Program of China (2018YFD0100905), the Oil Crops Research Institution Basal Research Fund of the Chinese Academy of Agricultural Sciences (CAAS), China (1610172018010), the major science and technology project, Ministry of science and technology, China (2018ZX08020001).

## Conflict of Interest

The authors declare that the research was conducted in the absence of any commercial or financial relationships that could be construed as a potential conflict of interest.
