# Supplementary material for: A Brassica napus Reductase Gene Dissected by Associative Transcriptomics Enhances Plant Adaption to Freezing Stress
Source: Front Plant Sci. 2020 Jun 26;11:971. doi: 10.3389/fpls.2020.00971 (PMC7333310; doi:10.3389/fpls.2020.00971)

Supplementary Figure S1. Temperature record during phenotypic investigation of the association panel. Blue line and green line represent the minimum and maximum temperature after 1st December 2016. The black and red lines represent 60 d of seedling stage with normal sowing date and late sowing date. Red arrows represent the daily low temperature during the phenotype investigation of association panel in the field.

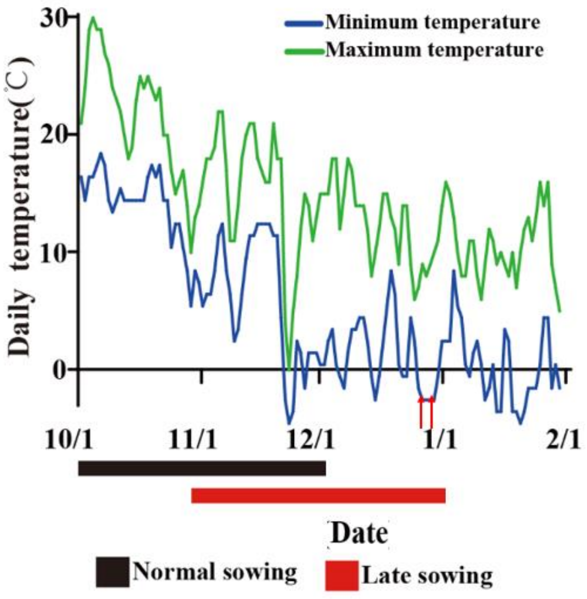

Supplement: Supplementary file 9 [file DataSheet_1.pdf]
