# Supplementary material for: A Brassica napus Reductase Gene Dissected by Associative Transcriptomics Enhances Plant Adaption to Freezing Stress
Source: Front Plant Sci. 2020 Jun 26;11:971. doi: 10.3389/fpls.2020.00971 (PMC7333310; doi:10.3389/fpls.2020.00971)

Supplementary Figure S2. Phenotype investigation of six rapeseed accessions under LT stress conditions. Performance of six rapeseed accessions before and after freezing treatment (-2 ℃ for 2 h). Scale=5 cm. Accessions 1~6 represented Sv706118/BnASSYST-378, Kajsa/BnASSYST-338, Callypso/BnASSYST-318, Libritta/BnASSYST-152, Gefion/BnASSYST-066, Jupiter/BnASSYST-148, respectively. Survival rates of six accessions after freezing treatment were cultivated based on three biological replicates. Normal represents 23 ℃, freezing treatment represents 4 h at -4 ℃.

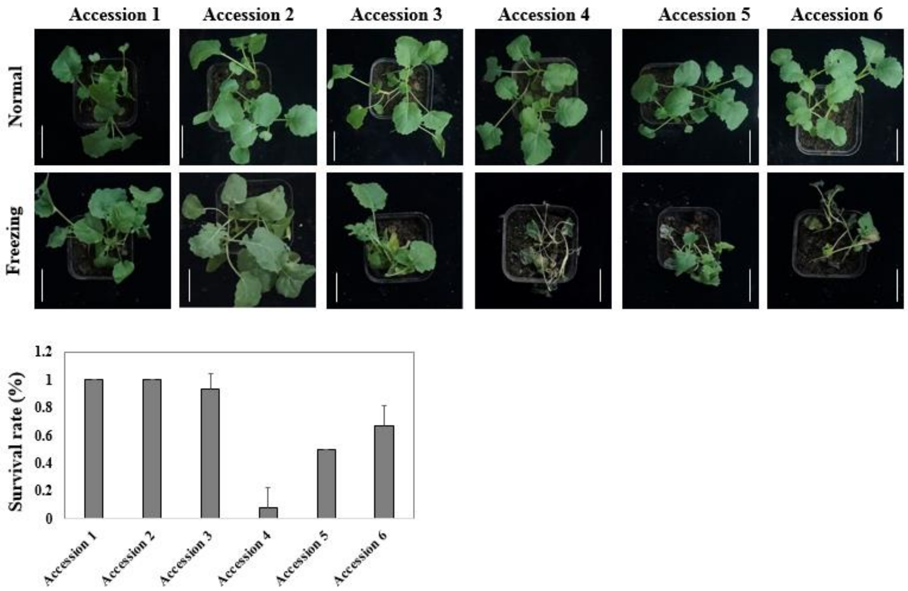

Supplement: Supplementary file 10 [file DataSheet_2.pdf]
