# Supplementary material for: A Brassica napus Reductase Gene Dissected by Associative Transcriptomics Enhances Plant Adaption to Freezing Stress
Source: Front Plant Sci. 2020 Jun 26;11:971. doi: 10.3389/fpls.2020.00971 (PMC7333310; doi:10.3389/fpls.2020.00971)

Supplementary Figure S3. Expression analysis of candidate genes. Changes in homolog genes of 22 candidate genes were investigated by qRT-PCR in six accessions under freezing conditions. Bars indicate the SE of three biological replicates. The rapeseed *ACTIN* gene is used as internal control. Accessions 1~6 represented Sv706118/BnASSYST-378, Kajsa/BnASSYST-338, Callypso/BnASSYST-318, Libritta/BnASSYST-152, Gefion/BnASSYST-066, Jupiter/BnASSYST-148, respectively. Normal represents 23 ℃, freezing treatment represents 4 h at -4 ℃.

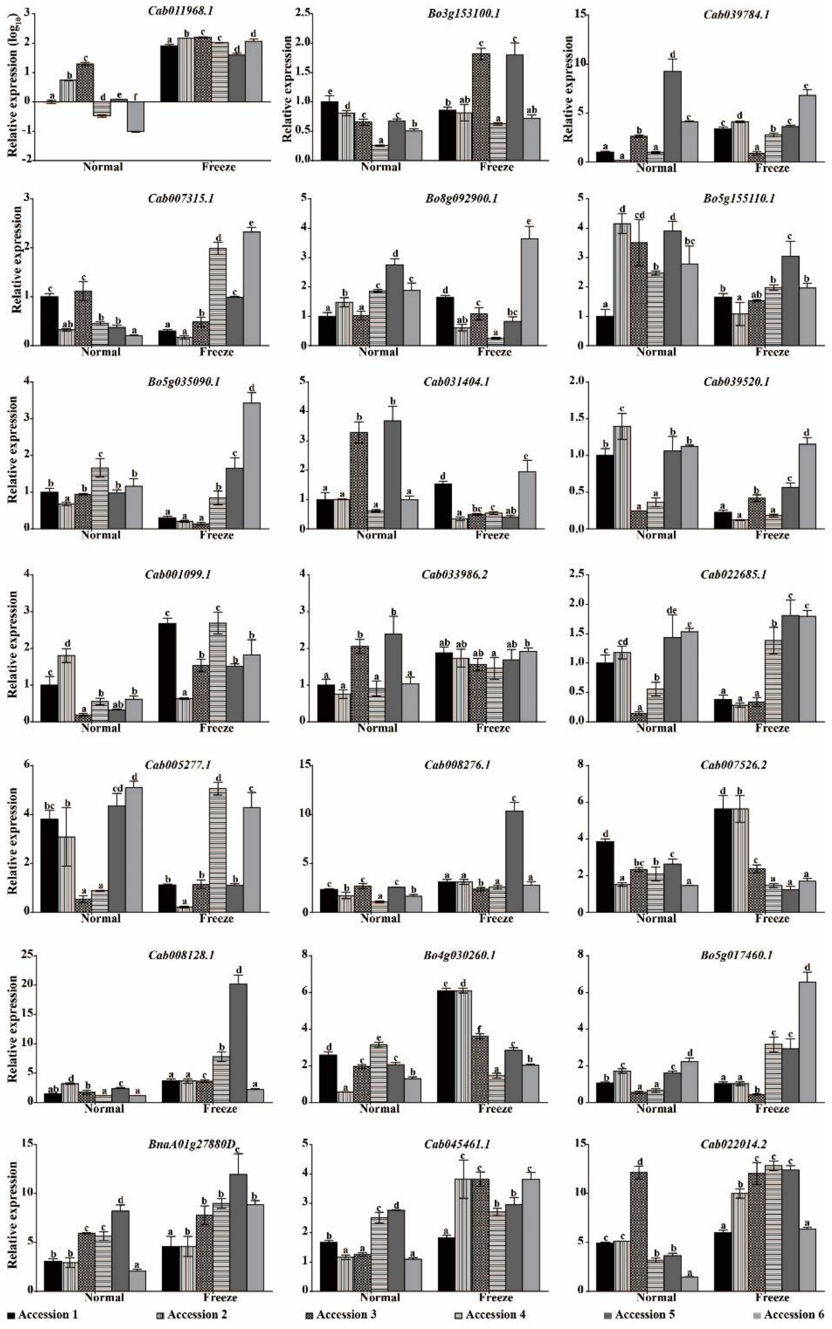

Supplement: Supplementary file 11 [file DataSheet_3.pdf]
