# Supplementary material for: A Brassica napus Reductase Gene Dissected by Associative Transcriptomics Enhances Plant Adaption to Freezing Stress
Source: Front Plant Sci. 2020 Jun 26;11:971. doi: 10.3389/fpls.2020.00971 (PMC7333310; doi:10.3389/fpls.2020.00971)

Supplementary Figure S4. Sequence alignment of BnTR1 and SDR proteins. The amino acids in red color represent the typical motifs (Gly-X_3-Gly-X-Gly), and a basic place (R49). The amino acids in grey represent the conserved residues that are responsible for catalytic function (N127, S155, Y168, K172, Y209).

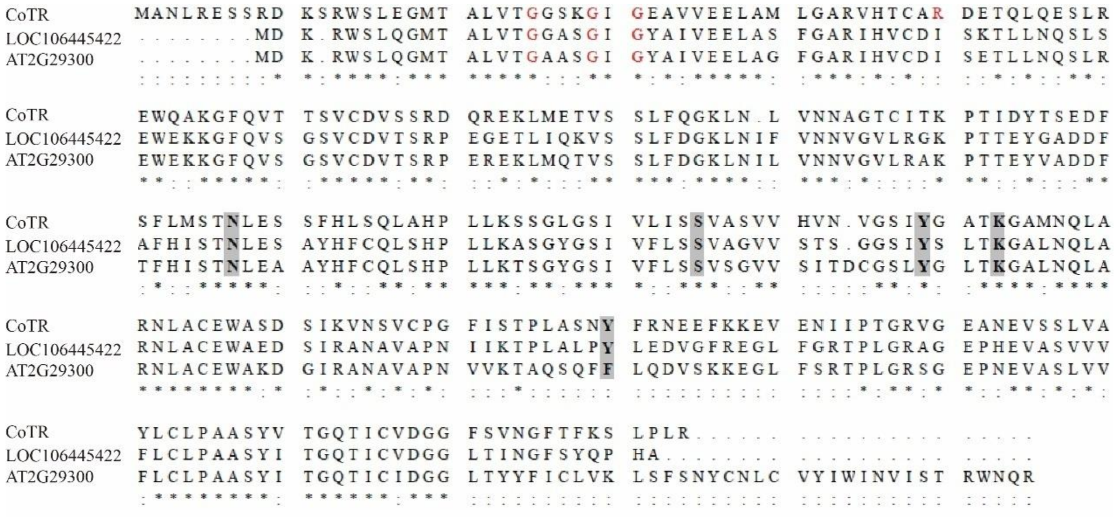

Supplement: Supplementary file 12 [file DataSheet_4.pdf]
