# Supplementary material for: A Brassica napus Reductase Gene Dissected by Associative Transcriptomics Enhances Plant Adaption to Freezing Stress
Source: Front Plant Sci. 2020 Jun 26;11:971. doi: 10.3389/fpls.2020.00971 (PMC7333310; doi:10.3389/fpls.2020.00971)

Supplementary Figure S5. Spatio-temporal and ubiquitous expression patterns of *BnTR1* in various tissues of Zhongshuang 11 (ZS11). Bars indicate the SE of three biological replicates. The rapeseed *ACTIN* gene is used as internal control.

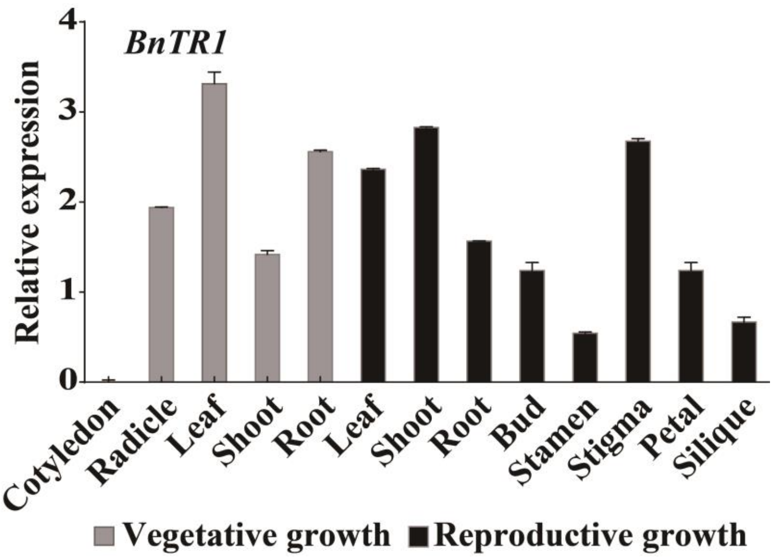

Supplement: Supplementary file 13 [file DataSheet_5.pdf]
