# Supplementary material for: A Brassica napus Reductase Gene Dissected by Associative Transcriptomics Enhances Plant Adaption to Freezing Stress
Source: Front Plant Sci. 2020 Jun 26;11:971. doi: 10.3389/fpls.2020.00971 (PMC7333310; doi:10.3389/fpls.2020.00971)

Supplementary Figure S6. Expression analysis of LT- or photosynthetic-related genes in *Arabidopsis* with atropine application. Changes in gene expression of *CBF1*, *CBF2*, *CBF3*, *COR15*, *RD29A*, *CAB1*, *CAB2*, *CAB3*, *CAB4*, *RAC*, *SPASE* in *Arabidopsis* WT plants with exogenous atropine application (0 nmol per plant, 10 nmol per plant, 30 nmol per plant) under freezing stress condition. Normal represents normal condition, freeze represents freezing stress (-4 ℃ for 4 h), recovery represents 3 d of recovery under normal condition. Bars indicate the SE of three biological replicates. The *Arabidopsis ACTIN* gene is used as internal control. Significant differences are determined by Student's *t* test (*$P< 0.05$, or **$P< 0.01$).

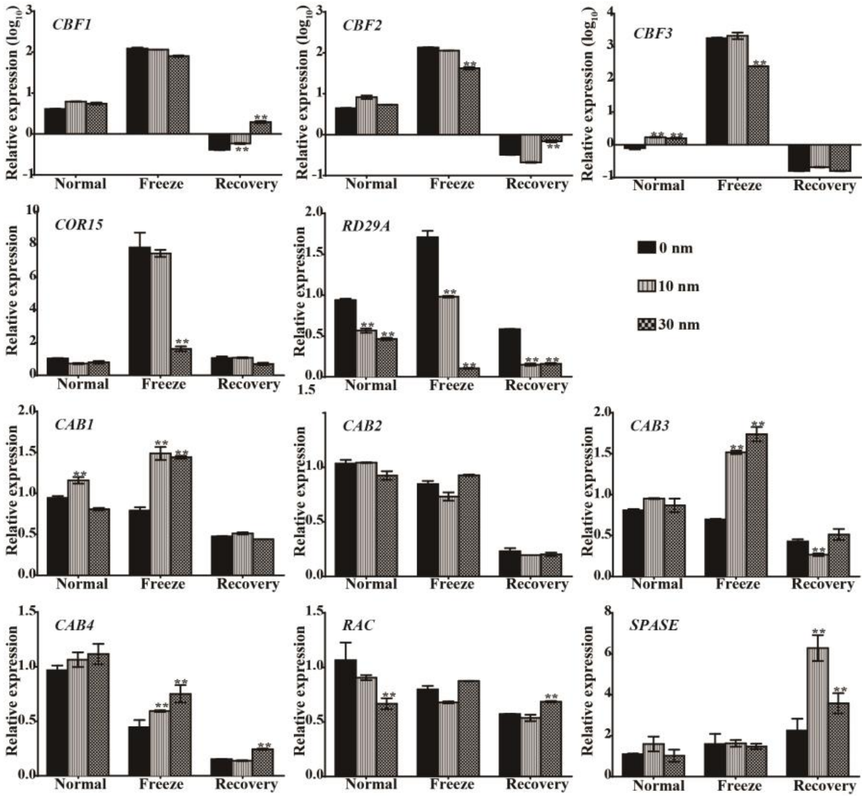

Supplement: Supplementary file 14 [file DataSheet_6.pdf]
